# Trialists perspectives on sustaining, spreading, and scaling-up of quality improvement interventions

**DOI:** 10.1186/s43058-021-00137-6

**Published:** 2021-04-01

**Authors:** Celia Laur, Ann Marie Corrado, Jeremy M. Grimshaw, Noah Ivers

**Affiliations:** 1 Women’s College Hospital Institute for Health System Solutions and Virtual Care, and Women’s College Research Institute, Women’s College Hospital, 76 Grenville Street, Toronto, Ontario Canada; 2 The Peter Gilgan Centre for Women’s Cancers, Women’s College Hospital, Toronto, ON Canada; 3 Clinical Epidemiology Program, Ottawa Hospital Research Institute, Ottawa, ON Canada; 4 Department of Medicine, University of Ottawa, Ottawa, ON Canada; 5 Department of Family and Community Medicine and Institute of Health Policy, Management and Evaluation, University of Toronto, Toronto, ON Canada

**Keywords:** Sustainability, Spread, Scale, Implementation, Learning health systems, Knowledge translation, Quality improvement, Diabetes

## Abstract

**Background:**

Quality improvement (QI) evaluations rarely consider how a successful intervention can be sustained long term, nor how to spread or scale to other locations. A survey of authors of randomized trials of diabetes QI interventions included in an ongoing systematic review found that 78% of trials reported improved quality of care, but 40% of these trials were not sustained. This study explores why and how the effective interventions were sustained, spread, or scaled.

**Methods:**

A qualitative approach was used, focusing on case examples. Diabetes QI program trial authors were purposefully sampled and recruited for telephone interviews. Authors were eligible if they had completed the author survey, agreed to follow-up, and had a completed a diabetes QI trial they deemed “effective.” Snowball sampling was used if the participant identified someone who could provide a different perspective on the same trial. Interviews were transcribed verbatim. Inductive thematic analysis was conducted to identify barriers and facilitators to sustainability, spread, and/or scale of the QI program, using case examples to show trajectories across projects and people.

**Results:**

Eleven of 44 eligible trialists participated in an interview. Four reported that the intervention was “sustained” and nine were “spread,” however, interviews highlighted that these terms were interpreted differently over time and between participants. Participant stories highlighted the varied trajectories of how projects evolved and how some research careers adapted to increase impact. Three interacting themes, termed the “3C’s,” helped explain the variation in sustainability, spread, and scale: (i) understanding the *concepts* of implementation, sustainability, sustainment, spread, and scale; (ii) having the appropriate *competencies*; and (iii) the need for individual, organizational, and system *capacity*.

**Conclusions:**

Challenges in defining sustainability, spread and scale make it difficult to fully understand impact. However, it is clear that from the beginning of intervention design, trialists need to understand the concepts and have the competency and capacity to plan for feasible and sustainable interventions that have potential to be sustained, spread and/or scaled if found to be effective.

**Supplementary Information:**

The online version contains supplementary material available at 10.1186/s43058-021-00137-6.

Contributions to the literature

- Quality improvement evaluations rarely consider how their intervention can be sustained long term, nor how to spread or scale to other locations.
- Evaluating what happens to quality improvement programs shown to be effective in randomized trials after their research funding is completed is a novel way to assess spread, scale, and sustainability.
- To achieve their long-term goals, trialists, including implementation scientists and practitioners, need to (i) consider the key *concepts* relevant to sustainability, spread, and scale; (ii) develop the appropriate *competencies*; and (iii) leverage the necessary individual, organizational, and system *capacity*.

## Background

Quality improvement (QI) programs aim to reduce the gap between research evidence of optimal care and the current care provided by healthcare professionals [1]. The investments needed to design, implement, and evaluate QI programs can be more worthwhile if programs shown to effectively improve care are sustained—as a minimum in the local settings where initial implementation occurred [2], and ideally spread or scaled-up to new settings to increase impact.

Sustainability has been described as “one of the least understood and most vexing issues for implementation research” [3]. One contributing factor to this difficulty is the inconsistent use of definitions [3–7]. In a systematic review of 92 studies about how sustainability is conceptualized and measured in evaluations of healthcare improvement programs and interventions, 53 studies explicitly mentioned sustainability, yet only 27 studies provided a definition, and 32 definitions were used [8]. For our study, we used the Moore et al. (2017) [6] definition of sustainability: “(1) after a defined period of time, (2) the program, clinical intervention, and/or implementation strategies continue to be delivered and/or (3) individual behavior change (i.e., clinician, patient) is maintained; (4) the program and individual behavior change may evolve or adapt while (5) continuing to produce benefits for individuals/ systems” [6] (p.6); and the Shaw et al. (2018) [9] definitions of spread and scale with a main difference being that spread is for “complex” problems where following a specific formula may not work and extensive adaptation may be needed. Scale is for “complicated” problems where formulas are critical and a high level of expertise is needed, yet solutions may not need to be adapted [9, 10].

Along with consistent definitions, theories, models, and frameworks (TMFs) are important to guide the design and analysis of interventions. In considering sustainability, the Dynamic Sustainability Framework involves “continued learning and problem solving, ongoing adaptation of interventions with a primary focus on fit between interventions and multi-level contexts, and expectations for ongoing improvement as opposed to diminishing outcomes over time” [2] (p. 1). The Greenhalgh et al. (2017) “Nonadoption, Abandonment, Scale-up, Spread, and Sustainability” (NASSS) Framework [11] is also frequently used as it provides domains to consider in the adoption of a new technology. More broadly, the Consolidated Framework for Implementation Research (CFIR) brings together key constructs across five domains (intervention characteristics, outer setting, inner setting, characteristics of individuals, and process) and is associated with effective implementation [12]. The Exploration, Preparation, Implementation, Sustainment (EPIS) Implementation Framework can also be used as a guide for effective implementation, focusing on four phases, including exploration, preparation, implementation, and sustainment [13].

The need for effective QI programs to be sustained, spread, and/or scaled-up is self-evident; however, there is minimal research in this area [2–4, 8, 14–16]. As diabetes is a chronic disease with high impact in terms of health care resource utilization, costs, societal impact, and health outcomes [17–19], more consideration for how to continue effective diabetes QI programs is needed. An ongoing systematic review of diabetes QI intervention trials conducted a sub-study of included trials (*n*=226) published between 2004 and 2014 [1]. In this sub-study, an author survey (*n*=94 responses) found that 78% (73/94) of trials observed improved quality of care, but 40% (29/73) were not sustained following trial completion. QI programs were reported as sustained in 19% (4/21) of trials where no improvements in quality of care were achieved [20]. The aim of this study was to follow up from these survey results to explore trialists’ experiences with sustaining, spreading, and scaling-up of QI programs that effectively improved care for people living with diabetes, after termination of initial funding of the program.

## Methods

### Study design

A qualitative approach was taken, focusing on specific case examples. Cases were selected based on results from a previously conducted survey of trial authors. That survey sampled a sub-set of trials (those published between 2004 and 2014) included within a larger systematic review of randomized trials of diabetes QI interventions [1, 20]. The survey results informed recruitment and interview questions for one-to-one telephone interviews with authors. A case-based approach was taken to gain insights from authors who were considered information rich from their direct experience with QI interventions that improved care for patients living with diabetes [21, 22]. The publications and survey results were used to provide a comprehensive view of each case.

### Eligibility, recruitment and sample size

Recruitment of eligible participants was conducted based on responses to the trial author survey [1]. In that survey study, first or corresponding authors of 226 trials published between 2004 and 2014 on diabetes QI program were e-mailed in 2018 and requested to complete an electronic survey about the perceived sustainability and spread of their intervention. The online survey was completed for 97 trials [20]. Of these 97 trials, 45 authors, representing 59 unique trials, agreed to be contacted for a follow-up interview.

Only authors who indicated their trial was effective, and thus suitable to be sustained and spread, were recruited for an interview. There were no restrictions on country of origin, however participants were required to speak English. One participant was recruited through snowball sampling, as during interviews, participants could identify other authors who they thought would provide a different perspective to the same trial.

Based on these criteria, 44 trialists (representing 49 studies) were eligible. These 44 trialists were recruited via email with a follow-up reminder e-mail sent after two weeks, with a third reminder e-mail sent to participants from non-American locations. Each participant who completed the interview was offered a $40 (Canadian) honorarium.

### Data collection

One-on-one telephone interviews were conducted by a trained interviewer (CL), following a semi-structured interview guide which was piloted with the first participant (Additional file 1). The interview guide was informed by validated frameworks including the Dynamic Sustainability Framework [2] and the NASSS framework [11]. Interviews were tailored to the participants based on details from their published trials [1] and their individual survey responses [20]. The interview focused on the index trial report included in the systematic review, however, participants were encouraged to discuss the work that led and followed the main paper in order to provide a comprehensive description of the trajectory of the work. No repeat interviews were conducted.

Basic demographic information was collected including participants’ gender, country, clinical, and current roles, if role is different than when the study was conducted, and current involvement in diabetes research. Written or verbal consent was obtained prior to the interview. All interviews were audio-recorded then transcribed verbatim by an external third party and identifying information was redacted. Context and reflection notes were taken by CL after each interview.

The interviewer was a female postdoctoral researcher with a background in implementation science and health services research, and a strong interest in sustainability, spread and scale. CL had no prior relationship with participants and explained her background before each interview.

### Analysis

Qualitative data from the interviews was analyzed inductively using NVivo 12 by two researchers (CL and AMC) using thematic analysis [23, 24] informed by a social constructionist paradigm [25] (p. 336–337), indicating that realities are shaped through our experiences and our interactions with others. Inductive thematic analysis was in keeping with our aims to interpret meaning from the interview data [25]. Coding of specific trial and demographic information was used to identified key elements within each of the cases. Career and project trajectories were explored inductively within each of the cases, showcasing the stories as well as the overarching themes. Case summaries were verified by the participants.

## Results

Eleven trial authors were recruited from the USA (*n*=8), Canada (*n*=2), and Australia (*n*=1), including physicians (*n*=5), pharmacists (*n*=2), non-clinicians (*n*=2), a dietitian (*n*=1), and a psychologist (*n*=1). Nine of 11 participants were male. Two participants were no longer involved in diabetes-related research (Table 1). One participant had two effective trial publications included, and two interviews were conducted for one trial. Interviews lasted between 20 and 61 min (average = 43 min).

**Table 1:** Demographic information of interview participants

Demographic Information	Participants, ***n*** (%)
**# of participants**	11
**Gender**	
Female	2 (18)
Male	9 (82)
**Country**	
USA	8 (73)
Canada	2 (18)
Australia	1 (9)
**Clinical role**	
Physician	5 (45)
Pharmacist	2 (18)
Dietitian	1 (9)
Psychologist	1 (9)
Non-clinical	2 (18)
**Current role**	
Professor	6 (55)
Implementer	1 (9)
Industry	1 (9)
Other	3 (27)
**Role is different than when conducted the study**	
Yes	4 (36)
No	7 (64)
**Still involved in diabetes research**	
Yes	9 (82)
No	2 (18)
**Years working in research**	
10–20 years	2 (18)
21–30 years	3 (27)
31–40 years	5 (45)
41–50 years	1 (9)
**Years working in diabetes**	
10–20 years	5 (45)
21–30 years	4 (36)
31–40 years	2 (18)
**Principal Investigator on the study**	9 (82)

### Sustained interventions

Four of the 11 studies reported in the survey that the intervention was “sustained”; however, interviews provided further insight into what was meant by sustainability. One participant clarified that only the ideas or documents were sustained, such as elements that authors thought would be effective with minor modifications, or the documents produced as part of the intervention were sustained, not the whole intervention. Another indicated it was sustained locally for a few years after funding ended but had since stopped, while another stated it was sustained locally in a new format, not what had been tested in the trial. Thus, only one of the interventions was actually sustained in a similar format at the time of the interview.

### Spread and scaled interventions

The survey indicated 9 of the interventions had spread. Interviews showed that the lead researcher was not necessarily involved in the spread, and that it was typically only some core components that were spread and not necessarily evaluated. One participant reported the intervention started spreading 5–8 years after their trial. Two trialists’ descriptions met the criteria for scale, using a top-down approach with minimal adaptation of what was used in the trial. One was stopped prior to full roll-out while the other was still being scaled.

Interview participants highlighted the challenge in asking yes/no questions about sustainability and spread. “The idea is that it works, yes or no, or it spreads, yes or no, that’s not the right question. I think the question should be where is it, and why is it, and then looking at the context.” (011). To further demonstrate these points, a comparison of original manuscripts, survey responses, and interview responses for effectiveness, sustainability, and spread is provided (Table 2).

**Table 2:** Comparison of responses between survey and interview results

Interview code	Discipline (role in research, if different)	Manuscript effectiveness	Survey: effective	Interview: effective	Survey: sustained	Interview: sustained	Survey: spread	Interview: spread
001 011 (same study)	Physician (Researcher) Researcher	✓	✓	X	X	X	✓	✓ (by another researcher)
002	Pharmacist (minimal research)	X	✓	X	X	X	X	X
003	Physician (minimal research)	✓	✓	✓	X	X	✓	Somewhat
004	Physician	X	✓	X	✓	Not sustained in original setting but made an impact 5–8 years later	✓	Spread 5–8 years later
005	Physician (Researcher)	✓	✓	✓	✓	✓	✓	✓
006	Pharmacist (Researcher)	✓	✓	✓	X	X	✓	✓ (Then stopped)
007	Dietitian (Implementer)	✓	✓	✓	✓	✓ (For a few years then ended)	✓	X Resources and ideas were spread
008	Physician (Researcher)	✓	✓	✓	✓	✓ (In a new format)	✓	✓ (In a new format)
009 (2 studies)	Business and Research	1 component effective	✓	X	X	Some ideas sustained	✓	✓ (The tool only)
		✓	✓	✓	X	X	X	✓ (Outside of research)
010	Psychologist (Research + Industry)	✓	✓	✓	X	X	✓	✓

### Trajectories

Given that the included trials were published between 6 and 14 years earlier, interviews focused on the trajectory of change over time for the intervention, ideas, and careers of the trialists. Case examples are provided in Table 3. One participant had developed an effective intervention and then left academia to implement it at scale. “One of the reasons I left my position as a research professor… is that I want in fact the things that I had shown worked and get them up to scale in the real business world.” (009). Another participant described the trajectory of the project that had an effective pilot that quickly gained interest and was scaled nationally alongside research to understand how it should be sustained and spread. However, the timing did not align, and the scaled interventions did not wait for the results about how to sustain and spread. Thus, the attempt at scaling the intervention failed for reasons later identified in the research. “The end result was that after the stage 2 [spreading the intervention] diabetes trial of the pilot program, the government discovered that they weren’t keen to continue the model as we had developed and implemented it.” (006). It is unclear if the barriers to scaling this intervention would have been overcome if the timing had allowed the research on sustainability and spread to be conducting ahead of the scaling-up.

**Table 3:** Select case examples to demonstrate trajectories within sustainability, spread, and scale of projects and careers

Case	Implementation intervention	Trajectory
**The challenges of scaling too quickly**	Diabetes self-management support delivered by community pharmacists.	The initial pilot of this self-management program showed benefit and led to a larger randomized control trial that demonstrated significant improvements in glycemic control, blood pressure control, and adherence in a number of outcomes. Based on this success, a plan was made to disseminate the program across the country in 2 stages. Stage 1 included a process evaluation of key barriers and facilitators to implementation and sustainability. Stage 2 was due to scale the program across the country, using the findings from stage 1. However, stage 2 was rolled out too soon, and the barriers identified in stage 1 could not be addressed or the facilitators applied before scaling began. As a result, the uptake in stage 2 was very low due to limitations in organizational capacity within the pharmacy as well as difficulties in identifying eligible people living with uncontrolled type 2 diabetes which was dependent on general practitioner confirmation. In the end, the government decided to fund a modified program that had limited evidence of effectiveness yet was more acceptable to the system.
**Pursuing the business case**	A computerized behavior and psychosocial assessment approach to support patients with type 2 diabetes	The initial research used motivational interviewing and a computerized behavior and psychological assessment tool to support patients living with type 2 diabetes. The research found that the motivational interviewing aspect was not effective, but the assessment tool was, as it used a patient-centered approach and helped clinicians support self-managed behaviors. A second Randomized Control Trial conducted with community health centers that served a diverse diabetes patient population demonstrated the patient-centered assessment was clinically effective (reduced HbA1c) in a team model. However, “there wasn’t a bridge of sustainability from the clinical research to real world scaling up and adoption.” The success of this project, but lack of sustainability, spread or scaling, is what led this researcher to leave his academic career in order to bring effective interventions into the real world. The tool developed in the research studies has now been converted into a sophisticated commercial product used by clinicians. By selling the product and relevant coaching, the revenue is used to do more research and grow the product. To achieve the population level impact that was needed, this researcher left academia in order to follow a strong business case to have the capacity to achieve impact and sustainability.
**Naïvely hoping for sustainability**	Development of shared medical appointments for diabetes and hypertension	The hospital was interested in how to deliver group interventions for chronic illness, and this researcher wanted to know if group interventions were cost-effective. The resulting Randomized Control Trial demonstrated that the group intervention was more effective than the control and was worth pursuing. This success led to the development of a manual for developing shared medical appointments, and a meta-analysis, that took 3 years, further demonstrated the success. With these positive outcomes, three new hospitals were asked to implement this intervention; however, the uptake was very low. The new hospitals found that the intervention was “too disruptive” and thus not implemented. Although the group intervention was evidence-based with proven effectiveness, due to challenges in implementation, a modified version was later implemented that was never fully evaluated. The researcher was not consulted about the modified version. On reflection, the researcher noted that they were naïve about the concepts, expecting that if the intervention worked and the team championed the effort, that others would “come along for the ride”—but they did not. The evidence-based version is no longer used, while the modified, less disruptive, intervention with a limited evidence-base is used.

### Key themes: concept, competence, and capacity

**Fig. 1:**
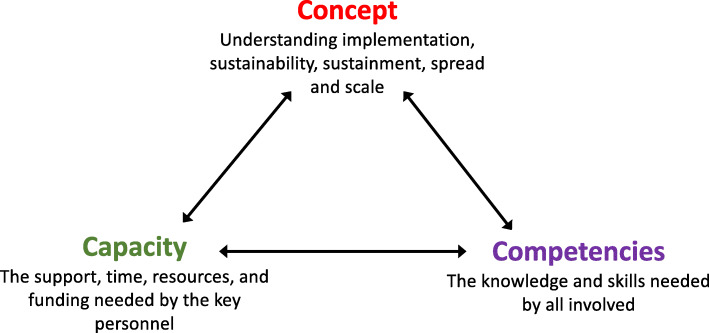
Interactions between the three main themes, concept, competence, and capacity

With these cases and trajectories, characteristics needed for sustainability, spread, and scale included three interacting themes, named the “3C’s” (Fig. 1): understanding the *concepts* of implementation, sustainability, sustainment, spread, and scale; having the appropriate *competencies*; and leveraging necessary individual and organizational *capacity*.

#### Concepts

Participants lamented that in hindsight they did not know how to consider issues related to feasibility, and which, if any, components of the interventions should be sustained, spread, or scaled. Since completing the initial trial, some participants had learned they needed to think beyond effectiveness if they wanted an intervention to continue.

“If you just think about doing a good study and putting it out there and putting the study out there in the literature and expecting it passively diffuse or for other people to just pick the ball up and run with it, I don't think that's likely to be very successful.” (001)

Feasibility of an intervention within various contexts needed to be considered, and many participants learned that enthusiasm and strong outcomes were not enough for sustainability, spread, or scale.

“We were naive at the time. We just sort of assumed that if I … use my energy and try to champion things that other people would get excited and come along for the ride and they didn’t.” (003)

Trialists highlighted the need to distinguish between related concepts relevant to sustainability, including the recognition that the processes needed to be sustained (sustainment) and the benefit continued (sustainability), as well as acknowledging that interventions should only be sustained if they continue to address the needs of the patients or organization. Participants also indicated the need to understand it may not be necessary or feasible for the whole intervention to continue after funding ends. Participants recognized that having core components of an intervention and ways to adapt to the context may be key facilitators.

“We have core intervention components that need to stay the same across sites but then some details that are modifiable for each site depending on what they view as what would work best for them.” (001)

However, when asked how core components were identified, the participant responded: “I don’t know. I’m not sure that we have codified it in a way where it's written down what is core and what’s not.” (001)

#### Competencies

Competencies needed to appropriately enact the concepts included being flexible, knowing how to build strong partnerships, develop a “maintenance” plan, and learn how healthcare works from a business perspective, in order to have a wider impact. Competencies required varied by role and level of involvement in the project; collaborating with the right people and organizations facilitated the development of these competencies within individuals and teams.

The competence to develop strong and lasting partnerships was key. There was concern that researchers who did not use this relationship approach would make collaboration more difficult for others. “We identify ‘helicopter researchers’ that come into a project and step out, and that’s a classic example of how to allow your research to fail and your community to become disenchanted.” (008). To put it another way. “Many researchers are able to use primary care practices like they use a Kleenex and pick up a study, and discard it when they're done, and go on to the next.” (008).

Participants also highlighted the need to consider early a long-term (maintenance) plan to determine the core aspects of an intervention that should continue, and how to achieve this, after project funding ended. Usually, this plan was not considered proactively within the original study and instead developed as a reaction to a newly identified need.

“We basically developed that [maintenance] protocol on the fly for indefinite engagement for as long as nurses felt they needed it. And it’s a lighter touch protocol with fewer calls, less involved calls, for that intervention, but a way to keep them engaged. And that is something that came out in the implementation setting that we hadn’t considered during the study.” (001)

Having the competence to know how to apply the maintenance plan, and how to engage stakeholders to establish buy-in with leadership and other champions were also facilitators. The benefit of knowing how to apply an “entrepreneurial approach” to spread and scale interventions was another frequently mentioned competency, particularly by American participants. In some cases, the trialists felt it was appropriate for them to be involved in spread and scale as an entrepreneur, understanding the business of healthcare and how to “commercialize”, yet many found this challenging or outside of their current remit.

“Our problem is, if we had a company, we could licence this to, or give to ourselves and sell it to them, they could market it, that way it would be in a lot more places. But I’m a primary care doctor, I’m a family doc, and I’ve got a full plate right now. Flying around the country talking to CEOs to sell something is not a part of my life, and it’s never going to be. I just don’t have the time. I could do it, but I don’t want to. So, we don’t have anybody out there pushing it actively, actively marketing it. We’re just busy developing it and trying to make it better, hoping that sooner or later somebody will pick it up and run with it.” (005)

In contrast to the emphasis on entrepreneurships and commercialization among American participants, those from other countries more often mentioned government support or involvement of specific public organizations with relevant expertise. However, participants agreed there was a need to understand perspectives outside of research to achieve their desired impact. This collaborative approach involved the competency of recognizing what was needed to make a good business case and the need to look outside of the research “bubble.”

“I would say definitely that there is a need for clinical researchers to disrupt themselves continuously. And if you stay in the research bubble, you’ll never get your solutions out to the marketplace unfortunately.” (009)

The skills to know when and how to stop (or de-implement) an intervention that was not working was another key competence. Although there were many reasons to stop an intervention, particularly if the intended benefit was not continuing, funding requirements and other pressures made it more difficult to know how to end an intervention without negatively impacting relationships or professional requirements.

“We talk about de-implementation. There’s a point where maybe the trial worked, you sustain it, but there’s a point where maybe it’s time to take it off or it doesn’t need to be sustained anymore. I’m not proposing necessarily that everything should be sustained, but I think some thoughtfulness in terms of knowing when to turn it on and when to turn it off needs to be considered.” (011)

#### Capacity

The capacity to build relationships between community partners and researchers was felt to be particularly crucial for initial implementation, with a need for role clarity after funding ended. Some participants felt a duty to support their community by building strong relationships with community partners. These relationships were about working together to benefit the community, organization, or system, so that if a project failed, everyone would learn from that and adapt or try something new since the relationship was not reliant on one project.

“Success is best defined by improvement in the delivery of care. I think it’s an ethical obligation of a researcher to continue to make sure that the communities that they work with see a benefit if there is benefit demonstrated in the research.” (008)

The capacity and role of everyone involved, including the principal investigators, community partners, public and private organizations, lived experience advisors (also called “patient partners”), and other health system stakeholders varied depending on their level of involvement. Individual capacity was a significant barrier to implementation among those that considered implementation as part of their role. Many participants did not have the time, funding, approval, or motivation to continue to remain involved in all projects, nor build a strong enough foundation that it could continue on its own. The lack of capacity among those who felt it *was* their role raised several questions about who to involve and about how to have an impact within an academic and funding system that seems to prioritize publications and conferences.

“I think most grants only have a dissemination section which may only say that I’m going to go publish the paper, present it at a national conference. I don’t think that’s adequate anymore.” (011)

Perspectives varied about the role of researchers in QI projects after funding ended, including the need, willingness, and capacity to stay involved. One participant felt that the researcher should not continue to be involved, as a strong foundation should already have been established, leaving it with the community partner to continue the intervention. “They [researchers] really don’t have a role. The responsibility for sustaining it belongs to the organization.” (007) In other cases, it was the lack of capacity of the researcher due to competing priorities that limited their continued involvement. Role clarity after funding ended, with particular consideration for the capacity of all involved was recommended, recognizing the need for flexibility based on changing priorities and capacity levels.

“I think that it’s very much up to the researcher [for how they stay involved] and I think that different researchers have different levels of interest in follow up roles. … On the one hand the person who brings in the funding, the principal investigator, can be an important advocate and champion for implementation going forward and if that’s what you want then that’s terrific. … The flip side of that though is that sometimes that’s not what we want to do. … At times what we want to do is do the science and move on and do more science. … I don’t feel as though I can impose upon investigators and tell them that it is absolutely their job to become champions for this intervention when they may perceive their job as to be someone who enumerates data rather than implementing interventions.” (003)

Few participants had been trained in implementation, either relying on other experts, or not taking on the implementer role if they felt it was outside their academic remit. One participant who did put more thought into a sustainability plan, mainly did so because it was part of the funder requirements. “One of the requirements for funding was the development of a sustainability plan, and so it did make us think about it.” (010) Within this funder driven approach, recognition that not all initiatives should be sustained, spread, or scaled is needed. An ineffective project needs the opportunity to stop, without going against funder requirements, while also supporting effective initiatives to continue.

Although emphasis was on individual capacity, organization, and system capacity was an overarching issue that was thought to enable or inhibit individual capacity. Although not discussed frequently, the capacity of the organization or system to accept new interventions needed to be seriously considered. “There’s only so much room in the health care system, so if you put something there and you sustain it, at the end of the day you’ve got to think about what you’re going to take away.” (011). This raised several questions about the need for de-implementation, system capacity, and how to develop systems that can accommodate or support regular change. Using a multidisciplinary approach, planning for specific roles, and knowing how those roles will change over time, were seen as facilitators to address individual, organizational, and system capacity.

### Interactions

How much a trialist understood about the concepts and their role impacted their level of involvement in putting knowledge into practice. Some participants saw implementation as part of their role and that implementation concepts need to be considered in most health research. “I think that dissemination should always be a priority. It should be built into the DNA of any research project. People should look carefully at the cost and feasibility of widespread implementation.” (010). Others disagreed. “Sustainability really is more of an issue for implementation and for health service deliveries. It sort of moves beyond primary research.” (006).

There was recognition of a disconnect between the concept and capacity of the people applying those concepts, particularly when there was not enough knowledge among researchers about how the healthcare system works from a business perspective. “There’s a big gap between what researchers know and what systems do.” (005). This disconnect was also mentioned by those who discussed the difficulty of making an impact within the academic system versus the healthcare system, particularly when they do not follow the same metrics. Several questions were raised about how to address this issue, balancing the need for academic and applied research.

“From the perspective of a researcher who is doing research each day, publishing, going to conferences, teaching, it looks as if you’re effective. But when you look at the metrics, they’re not effective. … It’s pretty clear that the traditional research approach including the translational research approach isn’t having an impact, but that’s not the metric.” (009)

Those who considered it part of their role as researchers to consider implementation recognised that their capacity did not always align with what was needed, particularly regarding spreading and scaling-up effective interventions. “We’re still running out of a research shop. We’d like to commercialise and get rid of it but so far, we haven’t found somebody to commercialise it.” (005). Others discussed a different way of thinking about what was needed for implementation work, that did not align well with structured research. “There was some giving up of controls that we had to do, so that it [the intervention] could continue and develop further.” (011).

Researchers and health system stakeholders had a lot to learn from each other to understand the concepts, and needed to work together to build effective interventions, building the competence within their capacity. This was further identified through discussion about who provided input to the projects and the impact it was having.

“One of the important steps that we made was involving our target audience in our clinical research. … We used real-world practices that deliver care. We didn’t create this in an artificial environment and then expect our practices to adopt it. I think that integration of your target audience, I think using real-world practices and real people. I think that doing this in a community interventional trial enhances a generalisability and the sustainability. When a system sees that its own practices have improved, it’s more likely to adopt the technology if it’s successful.” (008)

Ownership of the change from those most impacted was also seen as a crucial, with those involved needing to feel like they have the competency and capacity to continue.

“Building a partnership with the people who will run it and enabling those frontline staff and the process owners to really own it. So, I think the researchers cannot be the process owners. I think those who will run the program need to own it. And I think they need to have authority to make decisions. And I think the researchers basically have to stand aside and allow it to develop with the skills of those who are the process owners. I think the researcher’s role is informing with the evidence. And the role of the process owner is informing the experience and the feedback that is received directly from patients.” (007)

## Discussion

Understanding how to support implementation, sustainability, spread, and scaling-up of health-related interventions helps researchers, community partners, and other health system stakeholders achieve long-lasting, individual, organizational, and system-level impact. This study examined the varied trajectories of projects and people who sought to improve population health, and three interacting themes were found: (i) understanding the *concepts* of implementation, sustainability, sustainment, spread, and scale; (ii) having the appropriate *competencies* to apply the concepts; and (iii) having the individual, organisational and system *capacity* to enact a sustainability plan. Each of the cases also raised questions about how *concepts* were defined, and how to best develop *competence* and *capacity* to act upon shared understanding of those *concepts*. This work aligns with and may be complementary to existing frameworks, including EPIS [13], such that the bridging factors, inner setting, and outer setting factors align with the individual and system capacity to focus on sustainability, spread, and scale. By identifying various paths to population health impact, the cases raise questions regarding how and when researchers should take on a role similar to entrepreneurs, and/or partner with private enterprise or public organizations to achieve their goals.

### Challenges in defining concepts

Varying interpretations of what was meant by the concepts of sustainability, spread, and scale, highlighted the challenges in evaluating impact when definitions or targets are unclear and inconsistent. Participants indicated when they thought their intervention was sustained or spread, yet each defined it differently. This lack of clarity also highlighted the challenges in developing a broader understanding of strategies to support sustainability, spread, and scale. Further work to define these terms will support those developing interventions to follow targets that make sense for their program and align with the wider literature. Although many participants did not use implementation TMFs, the need to consider sustainability, spread, and scale from the beginning was evident, and use of TMFs may help to standardize interpretations. TMFs, such as EPIS or CFIR, can be used as guides so the concepts are applied from the beginning, and may allow for increased comparison. Following TMFs also increases potential to improve understand of concepts and increase competence in planning for sustainability, spread, and/or scale from the beginning. Although sustainability is included in many implementation TMFs, different strategies may be needed for sustainability, spread, and scale, yet the lack of consistency makes it difficult to study.

### Building competence and capacity

There is increasing recognition of the need to consider how to implement, sustain, spread, and scale-up effective interventions, yet a greater understanding of how to support and train those interested in having this impact is needed. Strategies to support this need can be at the individual, organizational, or system level and include supporting collaborations between academics, communities, public and/or private organizations, and policy makers.

Our findings indicate that researchers involved in implementation should be able to work with others using relevant TMFs, to develop long-term plans that balance potency and feasibility of an intervention, considering if/how to ensure that the benefit continues, not just the process, after funding ends. Similarly, for developing competence and capacity among researchers in public organizations, such as in a learning health system (LHS) [26], domains for training have been suggested such as systems science, research methods, ethics, improvement and implementation science, and engagement, leadership, and research management [27].

Minimal training opportunities are currently available to build implementation competencies, particularly related to knowledge translation and building capacity for partnership research [28]. Of the programs that are offered, few had been published, and of the nine found between 2000 and 2019, none had been rigorously evaluated [28]. Programs such as the Canadian Institutes of Health Research, Health System Impact Fellowship, which embeds graduate students into a health system organization to support the development of key competencies, have shown promise within public and private organizations [29].

### Organisation and system strategies

To address competency and capacity needs at the organizational and system levels, results focused more on private enterprise through commercialization and a business approach for spread and scale; however, these models appear better suited for the American context and may not be applicable in all countries. At the public organization or research program level, another option to support practice change is the development of implementation laboratories [30]. These laboratories involve close collaboration between health systems and research teams to meet applied and scientific goals to understand which interventions work better and why, and to support implementation at scale [30].

LHSs are a potential system approach to address competency and capacity while working towards high value healthcare. LHSs are “dynamic health ecosystems where scientific, social, technological, policy, legal and ethical dimensions are synergistically aligned to enable cycles of continuous learning and improvement to be routinised and embedded across the system, thus enhancing value through an optimised balance of impacts on patient and provider experience, population health and health system costs.” [26]. A LHS connects organizational and system-level strategies through their six main pillars including, scientific, social, technological, policy, legal and ethical pillars, with some supports aligning with multiple pillars at the same time [26]. The core values of an LHS include accessibility, adaptability, cooperative and participatory leadership, governance, inclusiveness, person-focused, privacy, scientific integrity, transparency, and value in healthcare [31], and more recently equity, fairness, and solidarity [32, 33]. In order to achieve sustained, population-level impact, LHSs aim to encourage change processes that adapt to system need and are continuous over time [26, 34]. This aim is directly in line with the needs identified to support implementation, sustainability, spread, and scale.

A similar approach from the UK was the development of Collaborations for Leadership in Applied Health Research and Care (CLAHRCs), which included partnerships between universities and surrounding health service organisations. Their aim was “to create and embed approaches to research and its application that are specifically designed to take account of the way that health care is delivered across sectors and a clearly defined geographical area” [35]. Evaluations of CLAHRCs have focused on process measures; however, data on outcomes and impact is minimal [35]. In 2019, CLAHRCs were replaced with Applied Research Collaborations (ARCs) which were launched as partnerships between National Health Service (NHS) providers, universities, charities, local authorities, Academic Health Science Networks, and other organizations. The aim of ARCs was “to improve outcomes for patients and the public; improve the quality, delivery and efficiency of health and care services; and increase the sustainability of the health and care system both locally and nationally.” [36]. These strategies have the intention to support public organizational and system-level changes that encourage sustainability of effective interventions and population-level impact, yet many of them lack strong evidence of effectiveness.

To develop system-level strategies such as through LHSs, individuals and organizations need the appropriate support, training, and capacity. Specifically, academics need to be supported through applicable funding opportunities, recognition of impact in career advancement and tenure, and other strategies that support an increased focus on population-level impact. Community partners need further support to collaborate with implementers and academics through funding, resources, and increased time made available for implementing and sustaining improvement activities. LHSs will be difficult to implement without a strong understanding of the concepts of implementation, sustainability, spread, and scale, and the competence and capacity to apply those concepts.

### Strengths and limitations

Included trials were conducted up to 14 years ago, making it difficult for trialists to remember details. Although this is a limitation, the advantage of this approach was to see changes over time and explore project and career trajectories not typically available with short-term follow-up. The sample size was limited and although it aligns with the author survey results in terms of high American representation [20], results may focus on more American perspectives making them potentially less generalizable. For these reasons, this study proposes potential mechanisms based on these cases and raises questions for further exploration.

Although focused on diabetes interventions, participants spoke about experiences throughout their career, building on concepts relevant to diabetes and other work. As similar trajectories are seen in other fields, results are likely applicable beyond diabetes research. The interview guide was informed by the NASSS and Dynamic Sustainability frameworks, however, with the small number of participants, the unique trajectories of change for projects and people became the focus, with an emphasis on inductive rather than deductive coding.

Due to various interpretations and justification of sustainability and spread obtained by following-up on survey responses, it was difficult to determine if an intervention qualified as effective, sustained, spread, or scaled, including when developing Table 2. Attempts were made to compare survey and interview results to the initial publications for sustainability; however, whether or not the intervention was sustained was rarely mentioned since it likely would have been published before this was known. The survey did not ask if an intervention was scaled, which further limited the comparison.

### Next steps

When cases are considered in line with the development of LHSs, more focus is needed on understanding how adaptation is considered and monitored in sustainability, spread and scale. As mentioned in a systematic review by Movsisyan et al. (2019), knowing when to adapt, how, and to what extent is not straightforward [37], yet it is important in order to meet the new and/or changing context when sustaining, spreading and scaling effective interventions. Tools such as the Framework for Reporting Adaptations and Modifications-Enhanced (FRAME) [38] may be useful to understand and monitor adaptations. When considered with other literature [2, 8, 37, 39], this work leads to several questions including, what skills are needed to facilitate adaptation? With the need for a strong business case, how can researchers and community partners be supported to develop this case? How can we facilitate researchers to scale their effective interventions? How can those who want to follow through with the implementation or scalability process continue without being restricted by their academic pressures and institutions? How and when do we decide if an intervention should be continued, or not? Considering the relevant concepts, and how to ensure competence and capacity, will help answer some of these questions and further support effective interventions to have population-level impact. Learning from fields such as business and psychology may also provide a more fulsome perspective on how to implement these changes.

## Conclusion

Challenges in defining sustainability, spread, and scale make it difficult to fully understand the impact. However, it is clear that from the beginning of intervention design, trialists need to understand the concepts and have the competency and capacity to plan for feasible and sustainable interventions that have the potential to be sustained, spread, and/or scaled if found to be effective. In regard to these concepts, they also need to know which TMFs may be useful when designing, implementing, and evaluating their intervention, ensuring an understanding of these concepts is accompanied with appropriate competencies and capacity to make change. This study indicates opportunities for further clarity of concepts, ways to increase competencies and use of TMFs, and support from organizational and system structures to support capacity for the implementation, sustainability, and scaling-up of effective programs into the health system.

## Supplementary Information

**Additional file 1.** Interview Guide.

## Data Availability

To maintain confidentiality of participants, interview transcripts are not available for use.

## Abbreviations

ARC: Applied Research Collaboration
CLAHRC: Collaborations for Leadership in Applied Health Research and Care
LHS: Learning health system
NASSS: Nonadoption, Abandonment, Scale-up, Spread, and Sustainability
NHS: National Health Service
QI: Quality improvement
