# Supplementary material for: Trialists perspectives on sustaining, spreading, and scaling-up of quality improvement interventions
Source: Implement Sci Commun. 2021 Apr 1;2:35. doi: 10.1186/s43058-021-00137-6 (PMC8017766; doi:10.1186/s43058-021-00137-6)
Supplement: Supplementary file 1 — Additional file 1. Interview Guide. [file 43058_2021_137_MOESM1_ESM.docx]

**Interview Guide**

*Please note, questions were targeted based on the results of the author survey and the publications, as indicated in the square brackets.*

**Part A: Understanding the Program**

1. Please tell me about your study called [PAPER(S) TITLE]?

*Prompts:* Why that intervention? How did it get started? What was involved? How did it go?

1. You indicated the study was funded by [SURVEY RESPONSE]. Can you tell me more about that funding? Prompt: Source? Duration? Evaluation only? Both implementation and evaluation?
   1. What happened when the funding ended?
   2. Do you think it made a difference that the funding was for [IMPLEMENTATION ONLY, EVALUATION ONLY, BOTH]? How?
2. In the survey you mentioned that you [DID/DID NOT] think that your trial continued to be implemented in the clinical setting where you tested it. Can you tell me more about what you mean by that? Prompt: how do you know it continued/did not continue?
   1. If YES, what continued? How?
   2. If NO, why not?
3. In the survey you mentioned that you [DID/DID NOT] plan for sustainability. Can you tell me more about that? Prompt: what was your plan? Did you/ how did you carry it out?
4. Do you have any follow-up data from after the trial regarding the outcomes that the QI trial aimed to improve?
   1. If so, what data did you collect? How? How is it being used?
   2. If not, why not?

**Part B: Understanding who was involved**

1. What was your role as a researcher in this QI program? Prompt: Planning? Advising? Analysis?
   1. How did your involvement change after funding ended? Prompt: Still involved?
   2. What (would have) facilitated your continued involvement?
   3. What do you think is the role of a researcher after a QI project has ended?
2. Who else was involved in the QI program? Prompt: implementers, researchers, clinicians
   1. How were they involved?
   2. How did their involvement change after funding ended?
   3. How were local staff (clinicians, administrators) involved? How did this involvement change after funding ended?
   4. Were external facilitators involved? How? How did this involvement change after funding ended?
   5. Were individuals with lived experience (patients/family/care partners) involved? How? How did this involvement change after funding ended?
3. What was the composition of the team(s) involved in initial implementation? Prompt: gender, age, professions, expertise in KT, policy, economic evaluation?
   1. How do you think this team composition impacted your program?
   2. How did you think this team composition impacted your ability to sustain (or not sustain) the program?
4. What factors do you think had an impact on the ability to continue the QI program in the ORIGINAL setting? i.e., change in government, government policy, organisational policy, organisational structure
   1. How did these factors impact the ability to continue the QI program in a NEW setting(s)?

**Part C: Spreading and Scaling Up the Program**

1. In the survey, you indicated you [DID/DID NOT] implement [ALL/SOME/SIMILAR] of the program in a new clinical setting. Can you tell me more about what you mean by that?
   1. If YES:
      1. Can you talk me through how it started in the new setting(s)?
      2. Did the change spread from one location to the next, or was there more of a top-down influence in where it was implemented next? [DETERMINE IF SPREAD OR SCALE]
      3. Do you think this strategy was effective? Why?
      4. What would have facilitated uptake in new areas? Challenges?
      5. What was your role?
      6. Were any changes made to the QI program in the new setting(s)? Prompt: What changes? Why? Who was involved?
      7. Did you consider how ready a new setting was for this QI program? How?
      8. Are you doing anything to keep the QI program going in the new setting? If so, what?
      9. What do you think is the role of the researcher in bringing successful QI programs to new settings?
   2. If NO:
      1. Although your study improved patient outcomes, do you have any thoughts on why it may NOT have moved to other settings?
      2. What might have encouraged the program to be applied in a new setting?
      3. What do you think is the role of the researcher in bringing successful QI programs to new settings?

**Part D: Use of Theory**

1. Did you use any theories or frameworks to plan and implement your intervention? If not, why not?
   1. If YES: What frameworks did you use? How did you use them? Prompt: A guide?
   2. Did you use an sustainability frameworks
2. IF ADOPTED (sustained, spread, or scaled): Did you or the site use any theories or frameworks to guide how to continue and/or expand your intervention? If not, why not?
   1. If YES: What frameworks were used? By whom? Why? How were they used? Prompt: helpful? A guide?
3. Do you have any recommendations for others aiming to sustain or spread a QI program?
4. Do you have any final points about your trial or sustainability in general that we haven’t talked about yet?
5. Is there anyone else we should interview about this trial? If so, who and what is the best way to get in contact with them? [DECIDE HOW TO CONTACT]
